# Delta-Tocotrienol Suppresses Radiation-Induced MicroRNA-30 and Protects Mice and Human CD34+ Cells from Radiation Injury

**DOI:** 10.1371/journal.pone.0122258

**Published:** 2015-03-27

**Authors:** Xiang Hong Li, Cam T. Ha, Dadin Fu, Michael R. Landauer, Sanchita P. Ghosh, Mang Xiao

**Affiliations:** Radiation Countermeasures Program, Armed Forces Radiobiology Research Institute, Uniformed Services University of the Health Sciences, Bethesda, MD, United States of America; ENEA, ITALY

## Abstract

We reported that microRNA-30c (miR-30c) plays a key role in radiation-induced human cell damage through an apoptotic pathway. Herein we further evaluated radiation-induced miR-30 expression and mechanisms of delta-tocotrienol (DT3), a radiation countermeasure candidate, for regulating miR-30 in a mouse model and human hematopoietic CD34+ cells. CD2F1 mice were exposed to 0 (control) or 7–12.5 Gy total-body gamma-radiation, and CD34+ cells were irradiated with 0, 2 or 4 Gy of radiation. Single doses of DT3 (75 mg/kg, subcutaneous injection for mice or 2 μM for CD34+ cell culture) were administrated 24 h before irradiation and animal survival was monitored for 30 days. Mouse bone marrow (BM), jejunum, kidney, liver and serum as well as CD34+ cells were collected at 1, 4, 8, 24, 48 or 72 h after irradiation to determine apoptotic markers, pro-inflammatory cytokines interleukin (IL)-1β and IL-6, miR-30, and stress response protein expression. Our results showed that radiation-induced IL-1β release and cell damage are pathological states that lead to an early expression and secretion of miR-30b and miR-30c in mouse tissues and serum and in human CD34+ cells. DT3 suppressed IL-1β and miR-30 expression, protected against radiation-induced apoptosis in mouse and human cells, and increased survival of irradiated mice. Furthermore, an anti-IL-1β antibody downregulated radiation-induced NFκBp65 phosphorylation, inhibited miR-30 expression and protected CD34+ cells from radiation exposure. Knockdown of NFκBp65 by small interfering RNA (siRNA) significantly suppressed radiation-induced miR-30 expression in CD34+ cells. Our data suggest that DT3 protects human and mouse cells from radiation damage may through suppression of IL-1β-induced NFκB/miR-30 signaling.

## Introduction

We recently demonstrated that natural delta-tocotrienol (DT3), an isomer of vitamin E [1,2], significantly enhanced survival of mice after exposure to lethal doses of total-body irradiation (TBI), and protected mouse bone marrow (BM) and gastrointestinal (GI) tissue from radiation-induced damage through regulation of stress-response signal pathways involving Erk, mTOR and protein tyrosine kinase 6 [3,4]. Our data indicate that DT3 may have applications in protecting against radiation injury from emerging radiological and nuclear threats and radiotherapy-induced side effects to normal tissue.

Radiation causes cellular DNA damage leading to “danger signals” and antigen release. In addition, a massive radiation-induced pro-inflammatory factor release from injured cells may further result in activation of stress response signals and cell damage and depletion [5–10]. These signals and antigens can result in early radiation responses that affect the features of radiation injury in different animal tissues. The interleukin (IL)-1 family of cytokines are linked closely to the innate immune response and are the first line of host defense against stress-induced acute and chronic inflammation [11,12]. MicroRNAs (miRNA) are a class of small and noncoding RNA molecules (on average only 22 nucleotides long) found in eukaryotic cells. They have the ability to post-transcriptionally regulate gene expression via targeting the 3′ untranslated region (UTR) of messenger RNA transcripts (mRNAs) [13,14]. miRNA-mediated gene repression occurs through both translational repression and mRNA destabilization [15]. Mammalian genomes encode hundreds of conserved miRNAs that target mammalian genes and are abundant in many cell types [16]. miRNAs could regulate the cellular changes required to establish stress-induced cell damage phenotypes [17]. On the other hand, miRNA also can be regulated during its maturation process, from primary and precursor to mature miRNA [18], although the underlying mechanisms are not well understood.

We recently reported that radiation upregulates miR-30b and miR-30c in human hematopoietic CD34+ cells, and miR-30c plays a key role in radiation-induced human hematopoietic and osteoblast cell damage through negatively regulating expression of survival factor REDD1 (regulated in development and DNA damage responses 1) in these cells after γ-irradiation [19]. Our data also suggested that p53 and NFκB regulate REDD1 expression and the effects of REDD1 on survival of human cells after radiation exposure acted through suppression of stress response signals p21 and mTOR, and inhibition of the radiation-induced senescence and apoptosis in these cells [6,19]. In this study, we confirmed our previous *in vitro* results and extend our findings using an *in vivo* mouse model, to explore our hypothesis that the radioprotective effects of DT3 are mediated through regulation of miR-30 expression in irradiated cells. The levels of miR-30 in CD2F1 mouse BM, jejunum, kidney, liver and serum as well as human CD34+ cells were measured at different times after both sublethal and lethal doses of radiation and the effects and mechanisms of DT3 on miR-30 expression were evaluated.

## Materials and Methods

### Ethics Statement

Animals were housed in an Association for Assessment and Accreditation of Laboratory Animal Care (AAALAC)-approved facility at the Armed Forces Radiobiology Research Institute (AFRRI). All procedures involving animals were reviewed and approved by the AFRRI Institutional Animal Care and Use Committee (IACUC). Animals received total-body irradiation (TBI) in a bilateral gamma radiation field at AFRRI’s cobalt-60 (^60^Co) facility. Control animals were sham-irradiated and treated in the same manner as the irradiated animals, except the ^60^Co source was not raised from the shielding water pool. For the survival study, irradiated mice were monitored two to four times a day for clinical signs as described in the AFRRI-IACUC policy to categorize animals as morbid or moribund. When an animal met the definitive criteria for moribundity (abdominal breathing, inability to stand, or inability to right itself within 5 sec when placed gently on its side), it was humanely euthanized at an early endpoint using 100% CO_2_ inhalation followed by cervical dislocation, in accordance with the American Veterinary Medical Association (AVMA) Guidelines for the Euthanasia of Animals. For blood sample collection, animals were anaesthetized with 1–5% isoflurane in 100% oxygen using an anesthesia machine (Compac^5,^ Vet Equip In. CA), and blood samples were drawn by cardiac puncture. Animals were euthanized by cervical dislocation immediately after blood collection and tissue samples were then taken.

#### Mice

Twelve- to 14-week-old CD2F1 male mice (Harlan Laboratories, Indianapolis, IN) were used according to methods described in previous reports [4]. All animals were acclimatized upon arrival and representative animals were screened for evidence of disease. Animal rooms were maintained at 21± 2°C with 50% ± 10% humidity on a 12 h light/dark cycle. Commercial rodent chow (Harlan Teklad Rodent Diet 8604) was available *ad libitum* as was acidified water (pH = 2.5) to control opportunistic infections. Animals were chosen randomly for each experimental group.

#### Human CD34+ cells

Human primary hematopoietic CD34+ cells were provided by the Fred Hutchinson Cancer Research Center (Seattle, WA). Thawed CD34+ cells were cultured in serum-free medium consisting of Iscove’s Modified Dulbecco’s Medium (IMDM) supplemented with BIT 9500 (Stem Cell Technologies, Tukwila, WA) and penicillin/streptomycin. Recombinant human (rh) stem cell factor (SCF, 100 ng/ml), rh flt-3 ligand (FL, 100 ng/ml) and rh interleukin-3 (IL-3, 25 ng/ml) were added. All cytokines were purchased from PeproTech, Inc. (Rocky Hill, NJ). The CD34+ cells were incubated at 37°C with 5% CO_2_ [20].

### Administration of drug or vehicle

DT3 was purchased from Yasoo Health Inc. (Johnson City, TN). The drug was solubilized in saline with 5% Tween-80 that also served as the vehicle for the animal studies. DT3 (75 mg/kg) or vehicle was administered as a single subcutaneous injection aseptically at the nape of the mouse neck with a 23G needle, 24 h before radiation according to our previous report [4]. No infections or local reactions were noted at the site of injection. For the *in vitro* study, DT3 (2 μM) or vehicle was added to the human CD34+ cell culture 24 h before exposure to gamma-radiation.

### Radiation and survival study

The survival study consisted of two treatment conditions, vehicle and DT3. Mice in the vehicle-treated groups were irradiated with a single radiation dose of 8.5, 9, 9.5, 10, 10.5, or 11 Gy, and the DT3-treated groups received a single radiation dose of 10, 10.5, 11, 11.5, 12, or 12.5 Gy, at a dose rate of 0.6 Gy/min in the AFRRI ^60^Co radiation facility based on previous observations (N = 20/group) [21]. After irradiation, mice were returned to their home cages with food and water provided as usual. Survival was monitored for 30 days. The LD_50/30_ (lethal radiation dose that results in the mortality of 50% of mice in 30 days) for vehicle- and DT3-treated mice was calculated using probit analysis. Determination of the dose reduction factor (DRF) was calculated as the ratio of the LD_50/30_ radiation dose of DT3-treated mice to the LD_50/30_ radiation dose of vehicle-treated mice[22]. A previous unpublished experiment found no significant difference between the LD_50/30_ of mice treated with the vehicle compared to a saline-treated group. All surviving mice were euthanized at the completion of the observation period.

In separate experiments, cytokine and miRNA analyses were conducted in vehicle or DT3-treated mice tissues at 1, 4, 8, or 24 h after sham-radiation control, 7 Gy (sublethal dose) and 10 Gy (lethal dose) TBI (N = 6/time point). The protective effects of DT3 on the radiation-induced acute hematopoietic syndrome were evaluated in mouse bone marrow (BM) cells 24 h after 7 Gy TBI.

Human CD34+ cells were irradiated at doses of 0, 2 or 4 Gy (0.6 Gy/min) that had been previously determined to generate one and two logs of cell kill by clonogenic assay [20]. After irradiation, cells were washed once and cultured in fresh culture medium without DT3.

### Mouse serum and tissue preparation

At 1, 4, 8, or 24 h after TBI, mice were humanely euthanized for serum and tissue collection. The mice were deeply anesthetized prior to collecting whole blood through a cardiac blood draw in accordance with the approved IACUC protocol. Blood was transferred immediately to microtainer tubes (BD Microtainer Gold tube). Following 30 min coagulation at room temperature, the sera were well separated from the gel by 10 min-centrifugation at 10,000 × g/min, collected and stored at −80°C for later study. Once blood collection from individual mice and the euthanasia were completed, mouse tissues were collected. BM cells were collected from mouse femurs and humeri. After erythrocytes were lysed by erythrocyte lysis buffer (Qiagen GmbH, Hilden, Germany), total BM myeloid cells were collected for further experiment use. Mouse spleens, livers, kidneys, and jejuna were excised, rinsed with phosphate buffered saline (PBS), and snap-frozen in liquid nitrogen, then stored at −80°C for further use.

### RNA extraction and quantitative real-time PCR

Total RNA and miRNA from mouse cells and human CD34+ cells were extracted using mirVana miRNA isolation kits (Life Technologies) as reported previously [19]. miRNA from mouse serum was isolated using mirVana PARIS Kit (Ambion, Cat#AM1556) following the manufacturer’s protocol. Briefly, 150 μl of mouse serum was mixed with an equal volume of 2× denaturing solution and incubated on ice. miRNA was extracted with an equal volume of acid-phenol:chloroform, and the recovered aqueous phase was transferred into a fresh tube. 100% ethanol was added and the mixture was passed through a filter cartridge; the filter was washed with miRNA wash solution. miRNA was eluted with 100 μl 95°C nuclease-free water. RNA concentrations were determined by measuring OD on a NanoDrop spectrophotometer ND-1000 (Thermo Fisher Scientific, Waltham, MA) and total RNA quality was verified on the Agilent 2100 bioanalyzer (Agilent Technologies, Palo Alto, CA) with RNA 6000 Nano chips. Reverse transcription (RT) was performed using TaqMan miRNA RT Kits (Applied Biosystems, Foster City, CA) in triplicate according to the manufacturer’s instructions, and the resulting cDNAs of miR-30a,-30b, -30c, 30d and 30e were quantitatively amplified on an IQ5 (Bio-Rad) Real-Time PCR System. miRNA levels were normalized to U6 as an internal control.

### Protein extraction

The frozen mouse tissues or cultured human CD34+ cells were homogenized in 1× radio-immunoprecipitation assay buffer (RIPA, Sigma-Aldrich, St Louis, MO) (supplemented with a protease inhibitor tablet) by tissue homogenizer (Fast Prep-24, MP Biomedicals, Solon, OH), following manufacturer recommendations. After 15 min centrifugation at 12,000 × g/min, the supernatant was collected and protein concentrations were determined using a BCA assay kit (Pierce, Rockford, IL).

### Immunoblotting

The collected cell homogenates were denatured in Laemmli buffer supplemented with DTT (dithiothreitol), and the same amount of protein from each sample (100 to 120 μg) was loaded for SDS-PAGE electrophoresis. Subsequently, immunoblotting was performed following standard procedures with an enhanced chemiluminescence kit (Thermo Scientific, Rockford, IL). The images were captured by CCD camera and the resulting densitometry was assessed using ImageGauge software. Protein densitometry was normalized to beta-actin. Antibodies for p53, p21, NFκBp65, and phosphalate-NFκBp65 were purchased from Cell Signaling (Minneapolis, MN) and Santa Cruz (Santa Cruz Biotechnology, Dallas, TX), and beta-actin was obtained from Sigma-Aldrich (St Louis, MO).

### Cytokine quantitation by enzyme-linked immune sorbent assay (ELISA)

Quantitation of IL-1β and IL-6 was performed using ELISA kits suitable for detecting these cytokines in sera and cell lysates. Cytokine levels in mouse spleen homogenate were determined following assay instructions provided by manufacturers. Briefly, spleens from individual mice were homogenized and sonicated in PBS plus proteinase inhibitor, followed by 15 min of 12,000× g centrifugation. The supernatant was collected and subjected to protein determination (BCA assay). The supernatant with an equivalent amount of protein (10 to 100 μg) from each sample was evaluated in duplicate. ELISA kits were purchased from R&D Systems (Minneapolis, MN).

### Flow cytometry assay

Cell viability (trypan blue-negative cells) from all groups was calculated. Death and apoptotic markers and cell lineage-surface phenotypes were determined using BD FACSCaliber flow cytometry. All antibodies and dyes including anti-mouse lineage-antigens antibody, apoptosis marker Annexin-V, and 7-aminoactinomycin D (7AAD) as a cell death marker were purchased from BD Biosciences (San Jose, CA).

### Clonogenic assay

Clonogenicity of mouse BM cells and human CD34+ cells was quantified in standard semisolid cultures in triplicate using 1 mL of Methocult GF+ system for either mouse cells or human cells (StemCell Technologies) according to the manufacturer’s instructions, as described previously [20]. Briefly, mouse BM cells from pooled samples or CD34+ cells from liquid culture were washed twice with IMDM (Iscove’s Modified Dulbecco’s Media) and seeded at 1–5 × 10^4^ cells/dish (mouse cells) or 1 × 10^3^ cells/dish (CD34+ cells) in 35-cm cell culture dishes (BD Biosciences). Plates were scored for erythroid, granulocyte-macrophage, and mixed-lineage colonies after culturing for 10 days (for mouse colonies) or 14 days (for human colonies) at 37°C in 5% CO_2_.

### Modulation of miR-30 expression with IL-1β neutralizing antibody

Neutralization of IL-1β bioactivity was performed as described in the manufacturer’s instructions. Briefly, a neutralizing antibody (0.2 μg/ml) or control nonspecific IgG (both from R&D Systems) from the same species was added to the CD34+ cell culture with or without IL-1β (10 ng/mL, R&D Systems) treatment 1 h before being exposed to sham- or γ- radiation. IL-1β and the antibody were maintained in the cultures after radiation. Cells were used for quantitative real-time PCR to determine the effects of IL-1β neutralization on miR30 expression.

### NFκB siRNA transfection

NFκBp65 siRNA from siGENOME SMARTpool (Dharmacon Inc., Lafayette, CO) was transfected into CD34+ cells using a Nucleofector II (Amaxa Inc., Gaithersburg, MD) according to the manufacturer’s protocol. In brief, 10^6^ CD34+ cells were resuspended in 100 μl of human CD34 cell Nucleofector solution (Human CD34 cell Nucleofector Kit, Cat No. VPA-1003, Amaxa Inc.) with 1.5 μg of NFκBp65 siRNA-siGENOME SMARTpool and/or 1.5 μg of maxGFP siRNA (control provided in the siRNA Test Kit, Amaxa, Inc.). Samples were transferred into an Amaxa-certified cuvette and nucleotransferred with program U008 using Nucleofector II. After transfection, cells were immediately transferred into fresh, pre-warmed, cytokine-supplemented CD34+ culture medium and cultured in a 37°C incubator until control, IL-1β or IL-1β + anti-IL-1β treatment on the next day (24 h after siRNA transfection).

### Statistical analysis

Differences between means were compared by ANOVA and Student’s t tests. p<0.05 was considered statistically significant. Results are presented as means ± standard deviations or standard errors of the mean as indicated. To calculate dose reduction factors (DRF), probit analysis was performed on mortality data.

## Results

### DT3 protected mice from lethal doses of gamma radiation

**Fig 1:**
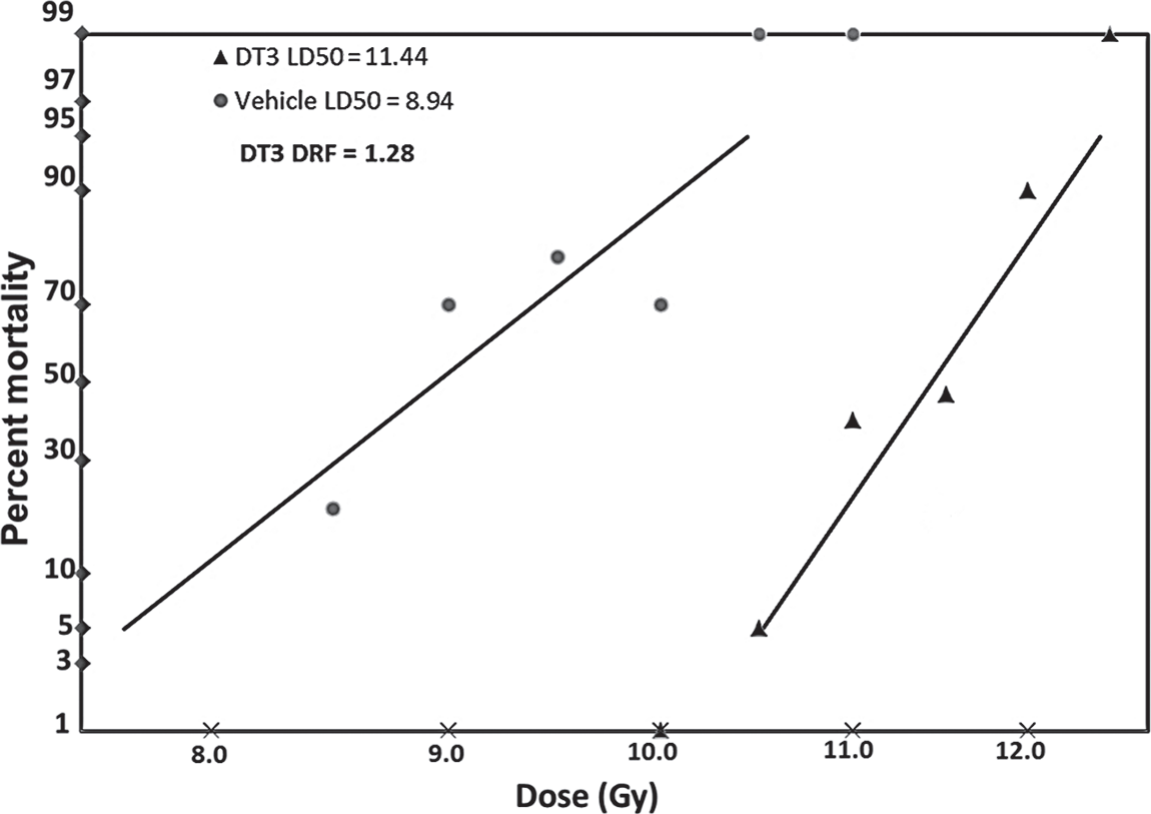
DT3 protected mice against lethal γ-irradiation. DT3 (75 mg/kg) or vehicle was administered as a single subcutaneous injection to mice 24 h before total-body gamma-radiation (TBI) at a dose rate of 0.6 Gy/min. Thirty-day survival was monitored after different doses of gamma-radiation. Survival data were used to evaluate the DT3 dose reduction factor (DRF, N = 20 mice/group). The DRF of DT3 was determined to be 1.28 (p<0.01).

DT3 (75 mg/kg) or vehicle was administered to CD2F1 mice 24 h before total-body irradiation (TBI) at various radiation doses as indicated in “Materials and Methods,” and 30-day survival data were used to evaluate the DT3 dose reduction factor (DRF, N = 20 mice/group) [23,24]. The probit lines in Fig 1 are drawn with percent mortality data. The LD_50/30_ radiation dose was 8.94 Gy for the vehicle-treated mice and 11.44 Gy for DT3-treated animals (p<0.01). The probit line for DT3-treated mice was significantly (p<0.01) shifted to the right compared with vehicle, yielding a DRF (95% confidence limits) of 1.28 (1.20–1.36). These data confirm that DT3 administration significantly protects mice from lethal doses of gamma-radiation [25].

### DT3 protected mouse and human hematopoietic stem and progenitor cells

**Fig 2:**
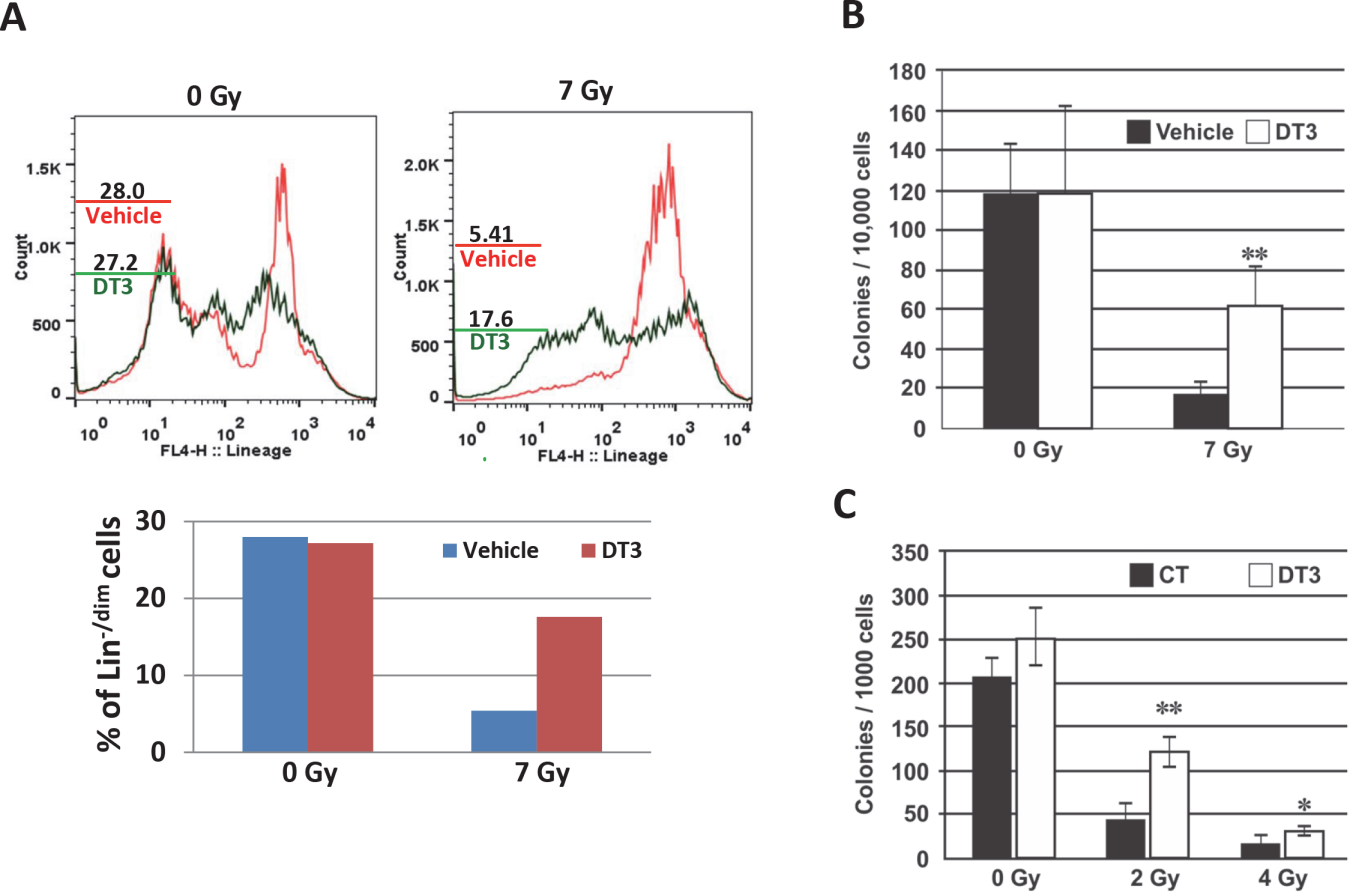
DT3 protected mouse and human hematopoietic stem and progenitor cells. Mouse bone marrow (BM) cells were collected from femurs and humeri 24 h after irradiation. (A) Flow cytometric analysis shows frequencies of live lineage-negative and lineage-dim (Lin-/dim) stem and progenitor cells in vehicle- or DT3-treated and sham or 7 Gy TBI mouse BM. Samples were pooled from 6 mice in the same group. Results were from one representative experiment of two independent experiments. (B) Clonogenicity of mouse BM cells was quantified in standard semisolid cultures in triplicate. Colonies were counted 10 days later. Results were from one representative experiment of two independent experiments (N = 6 mice /point/experiment). Means ± SD. **, p < 0.01, DT3-treated vs. vehicle-treated controls. (C) Clonogenicity of human CD34+ cells was quantified in standard semisolid cultures after 2 Gy or 4 Gy irradiation in triplicate. Colonies were counted 14 days later. Results were from one representative experiment of three independent experiments. Means ± SD. *, p<0.05, **, p < 0.01, DT3-treated vs. vehicle-treated controls.

Because hematopoietic tissue is very sensitive to radiation, we used a 7 Gy sublethal dose of TBI to evaluate the effects of DT3 on radiation-induced mouse hematopoietic tissue injury. BM cells were collected from femurs and humeri of vehicle or DT3-treated mice 24 h after 7 Gy irradiation. Total live BM myeloid cells from each mouse were measured by Trypan blue staining. BM samples from each group (6 mice/ group) were pooled for flow cytometric assays due to the limited number of BM cells collected from each mouse after irradiation. The frequency of radiation-induced BM hematopoietic stem and progenitor cell death were analyzed with anti-lineage antibody and 7-AAD co-staining. Lineage-negative and lineage-dim (Lin^-/dim^) population represented stem and progenitor cells and lineage-positive represented mature cells. Results from flow cytometric assays are shown in Fig 2A. In the 7AAD-negative alive cell population, radiation decreased Lin^-/dim^ hematopoietic stem and progenitor cells from 28% (vehicle-treated and sham-irradiated) to 5.41% (vehicle-treated and γ-irradiated). DT3 treatment protected stem and progenitor cells in marrow and resulted in 17.6% survival of Lin^-/dim^ population in irradiated mice. In addition, clonogenicity was compared between samples collected from individual mice treated with DT3 or vehicle 24 h post-irradiation. DT3 administration resulted in a total colony number increase from 18 ± 5 /10^4^ cells (vehicle-treated mice) to 60 ± 16 /10^4^ cells (DT3-treated mice) in 7 Gy irradiated samples (Fig 2B, p<0.01).

We further determined the survival effects of DT3 on γ-irradiated human hematopoietic CD34+ cells (*in vitro*). Consistent with the *in vivo* mouse study, addition of DT3 (2 μM) 24 h prior to γ-radiation markedly enhanced clonogenicity and resulted in 3- and 2-fold colony number increases in 2 and 4 Gy irradiated CD34+ cell samples, respectively (Fig 2C).

### DT3 repressed radiation-induced proinflammatory factor IL-1β and IL-6 expression in mouse spleens

**Fig 3:**
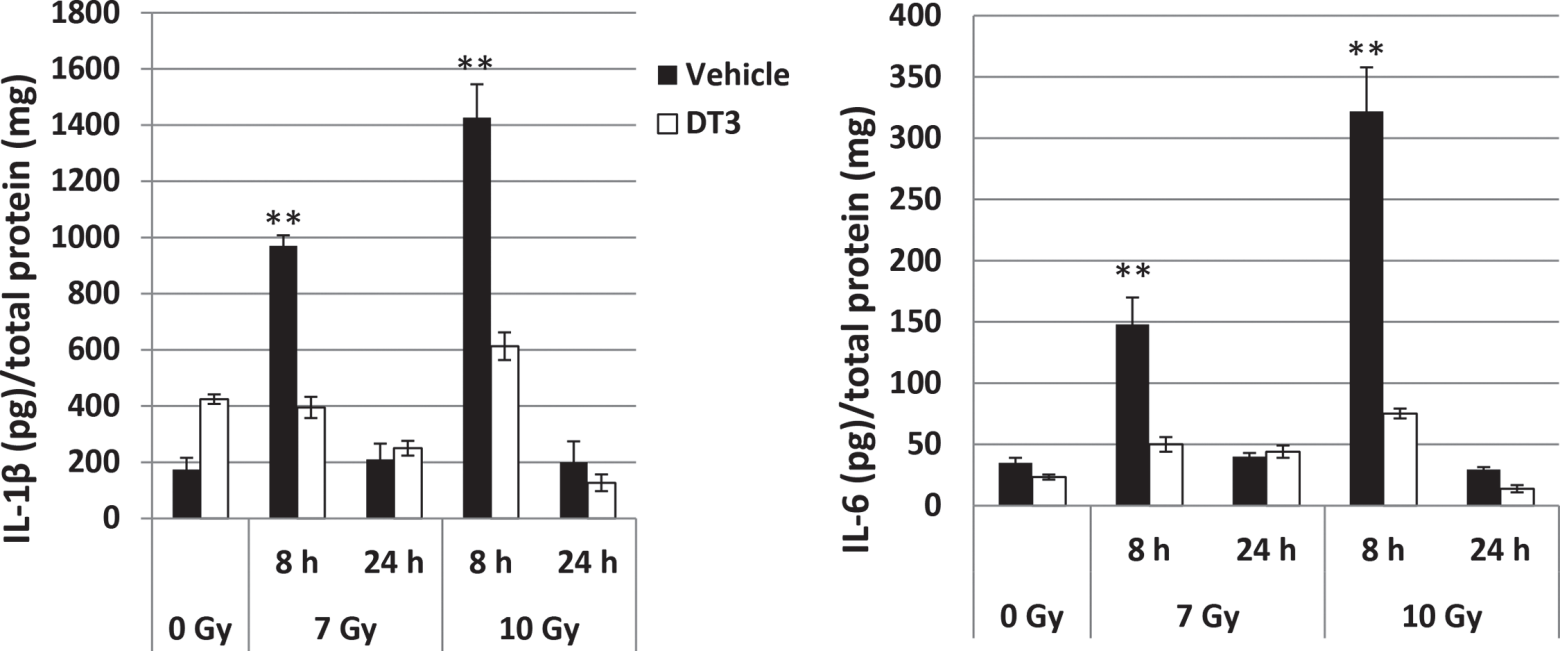
DT3 repressed radiation-induced proinflammatory factor IL-1β and IL-6 expression in mouse spleens. Mouse spleens were collected at 8 and 24 h after 0, 7, or 10 Gy TBI. Cell homogenates from spleen were generated in PBS after these organs had been collected. One mg of total protein from each sample in indicated groups (N = 6) was applied to determine IL-1β and IL-6 using ELISA. Results were from a total of two experiments (N = 12, 6 mice /point/experiment). Means ± SD. **, p < 0.01, DT3-treated vs. vehicle-treated controls.

Mouse spleens were collected at 8 and 24 h after 0, 7, or 10 Gy TBI and cell homogenates from spleens were generated in PBS. An optimized amount of total protein from each sample in indicated groups (N = 6/group) was applied to determine IL-1β and IL-6 using ELISA. These data are reported as cytokine levels detected in 1 mg of total protein/sample. Results in Fig 3 show that 7 and 10 Gy radiation induced more than 5-fold increases of IL-1β and IL-6 in spleen 8 h after irradiation compared to sham-irradiated control, and these increases were exhibited in a radiation dose-dependent manner. DT3 administration significantly inhibited the radiation-induced IL-1β and IL-6 expression in these cells. Levels of IL-1β and IL-6 reverted to baseline as shown in sham-irradiated controls at 24 h post-irradiation.

### DT3 downregulated radiation-induced miR-30 expression and secretion in mouse tissues and serum

**Fig 4:**
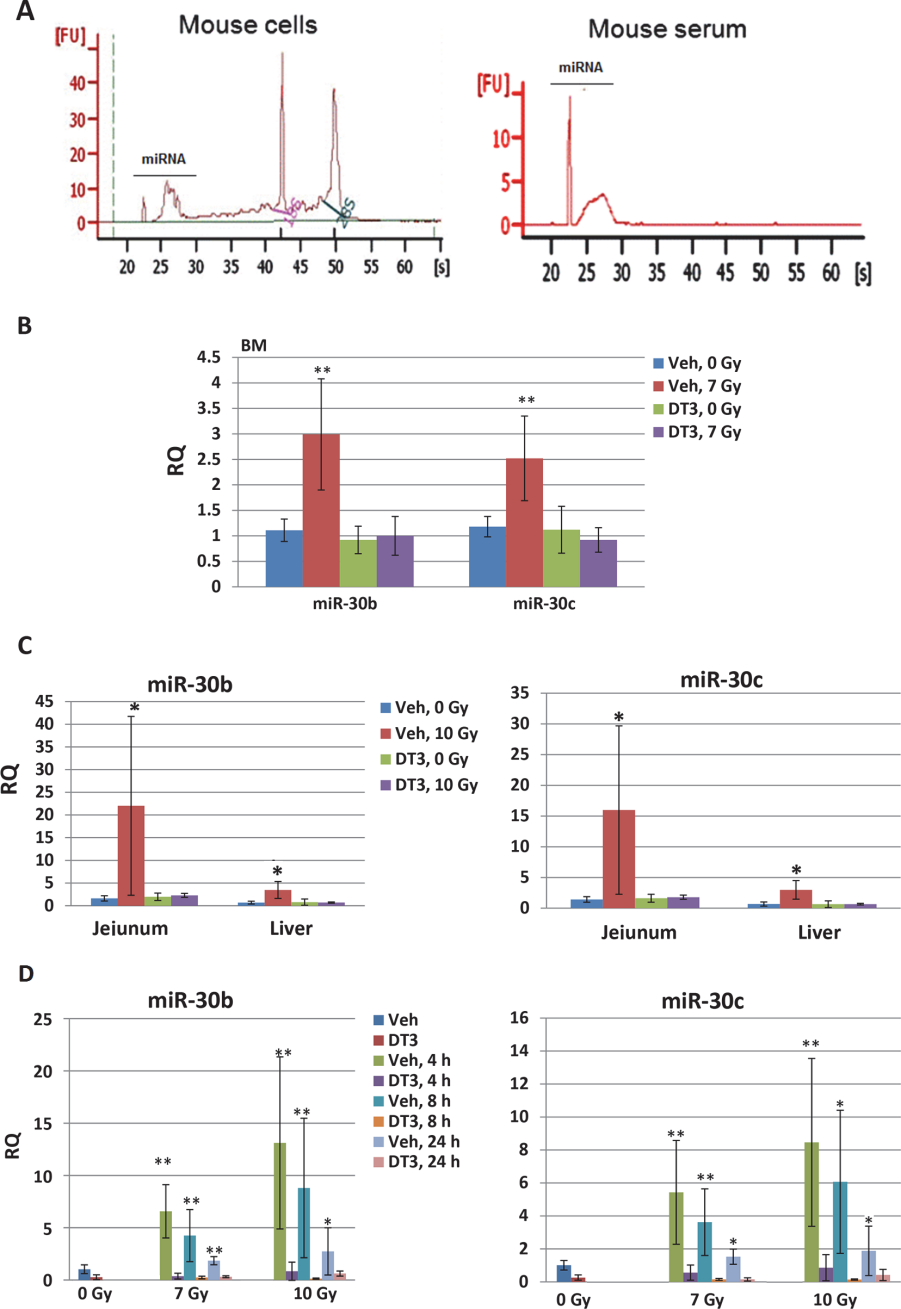
DT3 downregulated the expression and secretion of radiation-induced miR-30 in mouse tissues and serum. DT3 or vehicle was administrated 24 h before radiation and mouse tissues and serum were collected 1, 4, 8, or 24 h post-irradiation. (A) RNA and/or miRNA were extracted from mouse cells and serum using mirVana miRNA isolation kits following the manufacturer’s protocol. RNA quality was confirmed with an Agilent 2100 bioanalyzer (Agilent Technologies) with RNA 6000 Nano chips. DT3-treatment completely blocked the radiation-induced miR-30b and miR-30c expressions in mouse (B) BM cells after 7 Gy irradiation and in (C) jejunum and liver cells after 10 Gy irradiation, compared with vehicle-treated mice. (D) DT3-treatment suppressed miR-30b and miR-30c in 7 Gy and 10 Gy irradiated mouse serum at 4, 8, or 24 h post-irradiation. Results were from a total of two experiments, N = 6/group in each experiment; RQ = relative quantitation; * p<0.05, **p<0.01; mean ± SD, DT3-treated vs. vehicle-treated controls.

We previously demonstrated radiation-induced miR-30b and miR-30c expression in human hematopoietic CD34+ cells [19], and here we further examined the effects of gamma-radiation on miR-30b and miR-30c expression in mouse tissues. We asked whether DT3 can regulate expression of these miRNAs after radiation. DT3 or vehicle was administrated 24 h before irradiation and mouse tissues and serum were collected at 1, 4, 8, or 24 h post-irradiation. miRNAs were extracted from vehicle- or DT3-treated mouse BM, jejunum, kidney, liver and serum after sham- or γ- TBI at the indicated time points. The quality of total RNAs from mouse tissues and serum were verified using an Agilent 2100 bioanalyzer (Fig 4A). Using quantitative real-time RT-PCR assay, levels of miR-30b and miR-30c expression were determined in BM after 7 Gy, and also evaluated in jejunum, liver and kidney after 10 Gy because these tissues are relatively radiation-resistant and 10 Gy TBI can result in the acute gastrointestinal (GI) syndrome in CD2F1 mice [4]. We found that miR-30 was highly induced by radiation within 1 h in BM (Fig 4B), jejunum, and liver (Fig 4C), but not in kidney cells (data not shown). DT3-treatment completely blocked the radiation-induced expression of miR-30b and miR-30c in mouse BM, jejunum and liver cells compared with vehicle-treated mice (Fig 4B and 4C, N = 6/ group). In addition, levels of miR-30b and miR-30c in mouse serum were measured. Results in Fig 4D demonstrated the levels of miR-30b and miR-30c in serum changed in a radiation dose-dependent manner. Radiation induced both miR-30 subunits between 4–24 h after 7 and 10 Gy TBI. DT3 treatment suppressed miR-30b and miR-30c in irradiated mouse serum at all time points in comparison with vehicle-treated samples.

### DT3 or anti-IL-1β antibody suppressed radiation-induced miR-30 expression in human CD34+ cells

**Fig 5:**
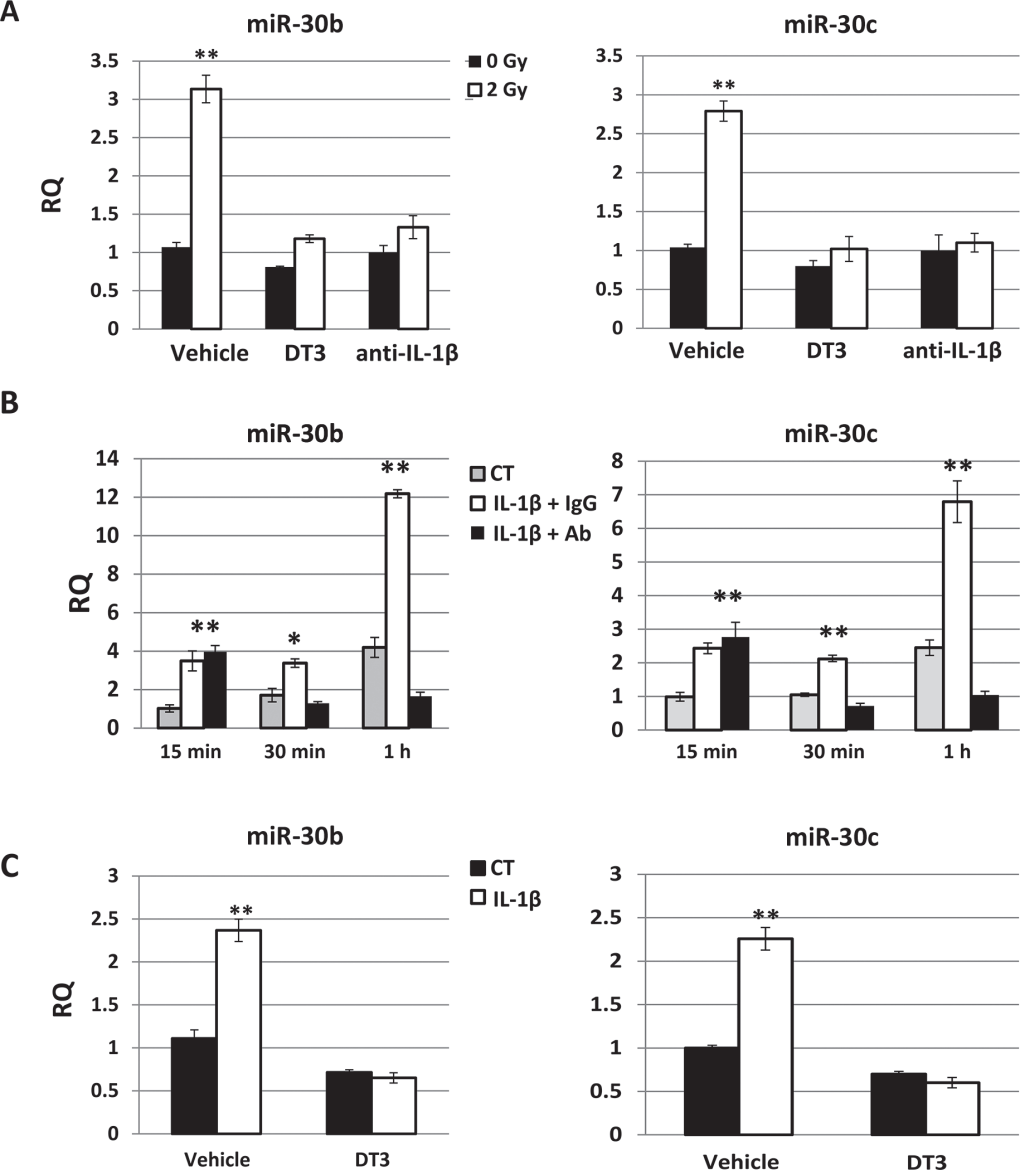
DT3 or anti-IL-1β antibody suppressed radiation-induced miR-30 expression in CD34+ cells. (A) Vehicle, DT3, or neutralizing antibody for IL-1β activation were added into CD34+ cell culture before 2 Gy radiation, and miR-30b and miR-30c expression were examined 1 h after irradiation. RQ = relative quantitation; **p<0.01; mean ± SD, 0 Gy vs. 2 Gy. (B) IL-1β (10 ng/mL) was added to CD34+ culture with anti-IL-1β antibody or nonspecific IgG control, and miR-30b and miR-30c expression were tested at 15 min, 30 min, and 1 h after addition of IL-1β. IL-1β significantly induced both miR-30b and miR-30c expression in CD34+ cells at all-time points. **p<0.01; mean ± SD, IL-1β+IgG-treated or IL-1β+ anti-IL-1β vs. vehicle-treated control. (C) Vehicle or DT3 was added to CD34+ culture 22 h before IL-1β treatment, and miR-30 expression was examined at 24 h after DT3 addition and 1 h after IL-1β treatment. DT3 administration abolished IL-1β-induced miR-30 expression in CD34+ cells. **p<0.01; mean ± SD, DT3-treated vs. vehicle-treated controls. Results were from total three experiments.

We next evaluated the effects of DT3 on radiation and/or IL-1β-induced miR-30 expression in human hematopoietic CD34+ cells because DT3 had suppressed the radiation-induced IL-1β and its downstream cytokine IL-6 production in mouse spleen (Fig 3) and jejunum [4]. Vehicle, DT3, or a neutralizing antibody for IL-1β activation were added into CD34+ cell culture before 2 Gy irradiation, and miR-30 expression was examined 1 h after irradiation. Fig 5A shows that 2 Gy radiation increased miR-30b and miR-30c by 3- and 2.5-fold in vehicle-treated CD34+ cell samples, respectively. Treatment with DT3 (2 μM, 24 h before irradiation) or an anti-IL-1β antibody (0.2 μg/mL, 1 h before irradiation) equally repressed expression of radiation-induced miR-30 in CD34+ cells. To determine whether the radiation-induced IL-1β increase contributed to miR-30 expression and whether DT3 could inhibit the miR-30 expression induced by IL-1β, we used assays to validate the effects of IL-1β on miR-30 expression in CD34+ cells (Fig 5B). IL-1β (10 ng/mL) was added to CD34+ culture with the anti-IL-1β antibody (0.2 μg/mL) or the same amount of a nonspecific IgG, and miR-30 expression was tested at 15 min, 30 min, and 1 h after addition of IL-1β. As expected, both miR-30b and miR-30c were expressed significantly in CD34+ cells 15 min after IL-1β addition and continually increased to 3-fold at 1 h after IL-1β-treatment as shown in Fig 5B (IL-1β + IgG). Addition of the anti-IL-1β antibody for 30 min completely neutralized the expression of IL-1β-induced miR-30 in these cells. Finally, vehicle or DT3 was added to CD34+ culture 22 h before IL-1β treatment, and miR-30 expression was examined at 24 h post-DT3 addition and 1 h after IL-1β treatment. Results shown in Fig 5C confirmed that DT3 administration abolished expression of IL-1β-induced miR-30 in CD34+ cells.

### Anti-IL-1β antibody protected CD34+ cells from radiation-induced apoptotic death

**Fig 6:**
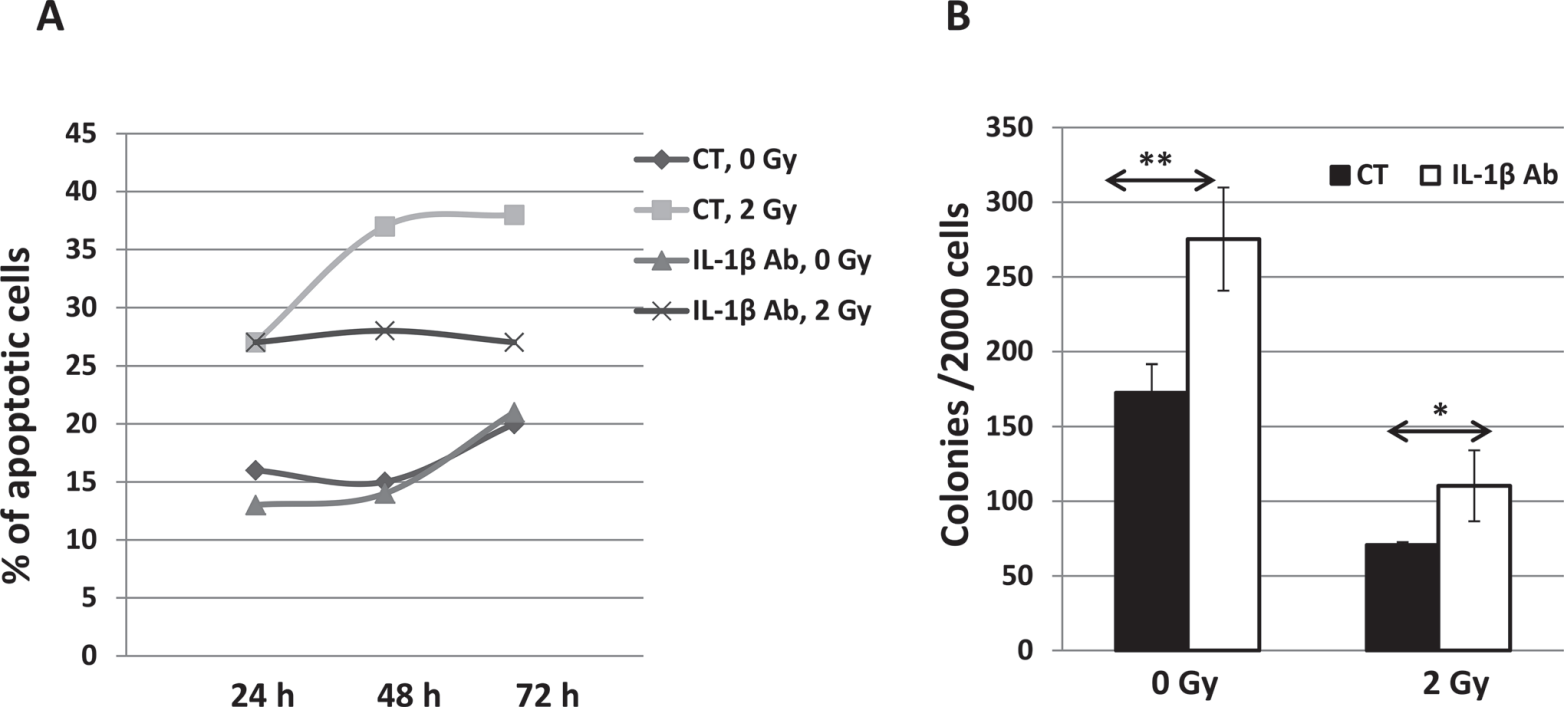
An anti-IL-1β antibody protected CD34+ cells from radiation-induced apoptosis. The anti-IL-1β antibody (0.2 μg/mL) or the same amount of nonspecific IgG as a control (CT) were added into CD34+ cell culture 1 h before sham- or 2 Gy radiation. (A) Cells were analyzed by flow cytometry with apoptotic and cell death marker Annexin-V and 7AAD staining at 24, 48, and 72 h after irradiation. Frequency of apoptosis (Annexin-V and 7AAD positive) cells is shown. (B) Clonogenic assay on survival of human hematopoietic progenitor cells was performed 1 day after irradiation. Colonies were counted at 14 days of culture. Colony generation was inhibited by radiation, and this inhibition was ameliorated by neutralization of IL-1β bioactivity using anti-IL-1β antibody. * p<0.05, **p<0.01; mean ± SD. Anti-IL-1β antibody vs. IgG control.

We reported recently that knockdown of miR-30c expression significantly protected CD34+ cells from radiation damage as shown by increased colony formation [19]. In this study, we further demonstrated the effects of DT3 and the anti-IL-1β antibody on suppression of radiation or IL-1β-induced miR-30 expression in CD34+ cells. DT3 protected clonogenicity of CD34+ cells after radiation exposure (Fig 2C). We sought to determine the efficacy of an anti-IL-1β antibody on survival of CD34+ cells after radiation. The anti-IL-1β antibody or a nonspecific IgG as a control, were added into CD34+ cell culture 1 h before sham- or 2 Gy irradiation. Cells were collected and analyzed by flow cytometer with apoptotic and cell death marker Annexin-V and 7AAD staining at 24, 48, and 72 h after irradiation. The frequencies of apoptotic cells are shown in Fig 6A: spontaneous cell death without radiation was 15–20% during cultivation, and 2 Gy irradiation enhanced cell death to 27% at 24 h, 37% at 48 h and 39% at 72 h post-irradiation. Addition of anti-IL-1β antibody protected against radiation-induced apoptotic cell death and resulted in a significant decrease of the frequency of apoptotic cells at 72 h post-irradiation. Clonogenic assays further confirmed the efficacy of the anti-IL-1β antibody on survival of human hematopoietic progenitor cells after irradiation (Fig 6B).

### NFκB activation was responsible for radiation-induced miR-30 expression in CD34+ cells

**Fig 7:**
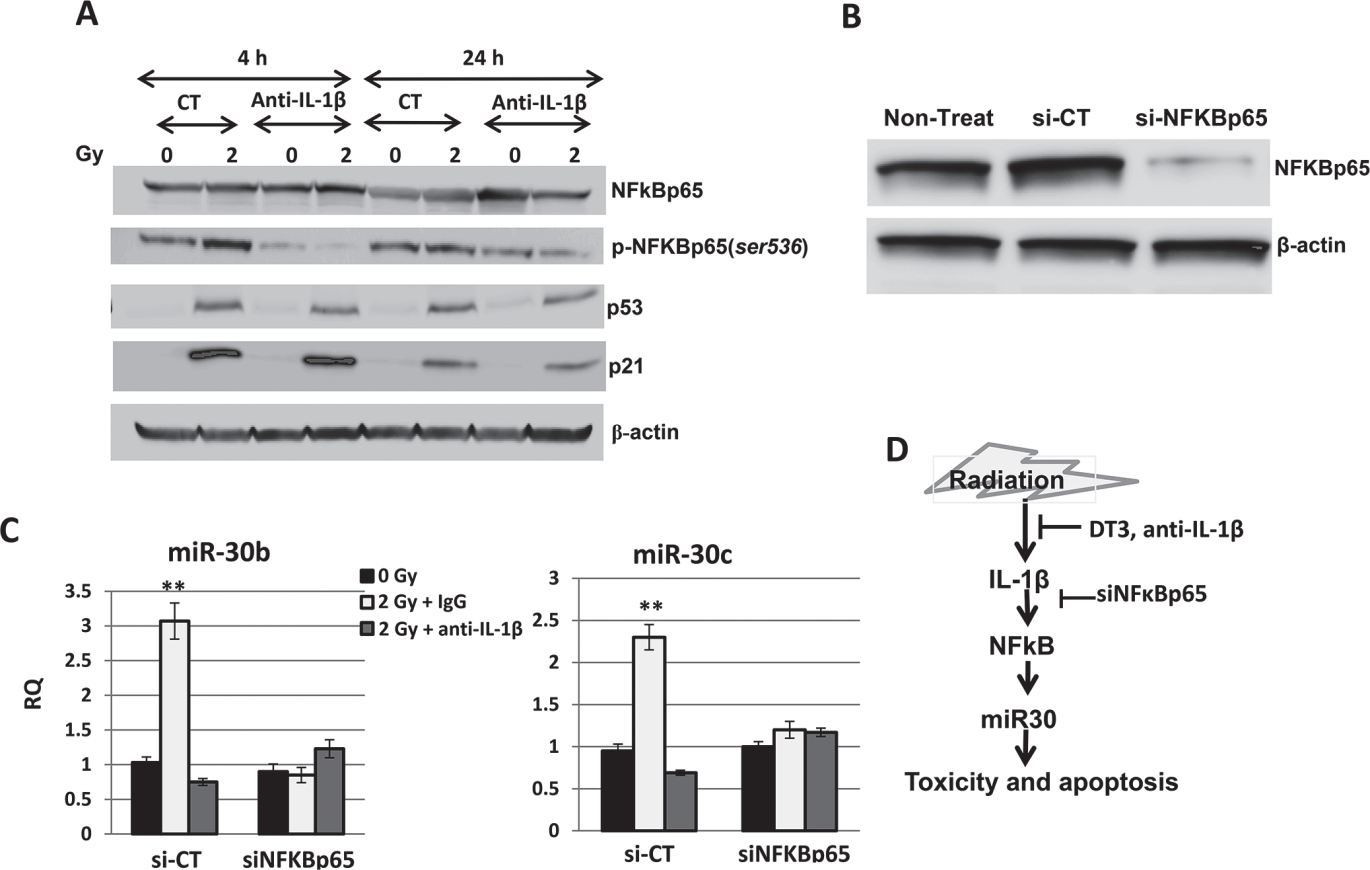
NFκB activation was responsible to radiation (and IL-1β)-induced miR-30 expression in CD34+ cells. (A) CD34+ cells treated with control or anti-IL-1β antibody were collected at 4 h and 24 h after 0 or 2 Gy irradiation. p53, p21, NFκB expression and phosphorylation of NFκB (ser536) were examined by immunoblotting assay with β-actin serving as an internal control. Addition of anti-IL-1β antibody before radiation resulted in a significant inhibition of NFκB phosphorylation in these cells at 4 h and 24 h post-irradiation. (B) Western blots show NFκBp65 and β-actin (loading control) expression in non-transfected, control-siRNA-transfected, and NFκBp65-siRNA-transfected samples. (C) MiR-30b and miR-30c expression were validated in sham (0 Gy) or 2 Gy irradiated with or without anti-IL-1β antibody-treated and siNFκBp65 or control-siRNA transfected cells by quantitative real-time RT-PCR. RQ = relative quantitation; results from three independent experiments, **p<0.01; mean ± SD. 0 Gy vs. 2 Gy. (D) Schematic diagram of the role of DT3 or anti-IL-1β antibody in radioprotection. DT3 or anti-IL-1β antibody inhibited radiation-induced IL-1β production and reversed IL-1β-induced NFκB/miR-30 stress signaling.

Radiation-induced IL-1β release from injured cells may further result in stress response signal activation and cell damage and depletion. To evaluate IL-1β-induced signal transduction in irradiated CD34+ cells, we examined expressions of stress-response factors p53, p21, and NF-κB, and their activation in γ-irradiated CD34+ cells with or without neutralization of IL-1β activity (Fig 7A). Data from the immunoblotting assay showed that p53 and p21 were highly expressed and phosphorylation of NFκB (*ser536*) was increased in nonspecific IgG control-treated CD34+ cell samples collected at 4 h and 24 h after 2 Gy irradiation compared with sham-irradiated samples. Neutralization of IL-1β activity resulted in a significant inhibition of NFκB phosphorylation in these cells at 4 h and 24 h post-irradiation, suggesting that radiation-induced NFκB activation in CD34+ cells is regulated by IL-1β. We further evaluated the interaction between radiation-induced IL-1β, NFκB activation and miR-30 in CD34+ cells. NFκB siRNA was transfected into CD34+ cells before sham- or 2 Gy with control or anti-IL-1β antibody treatment using Nucleofector technology as described under “Materials and Methods.” NFκB siGENOME SMARTpool (Dharmacon, Inc.) contains a mixture of four siRNAs targeting one human NFκBp65 gene, which silences gene expression at the mRNA level by at least 75%. Results from Western blot analysis (Fig 7B) showed that NFκBp65 protein levels markedly decreased after NFκBp65 siRNA transfection. In contrast, control siRNA-transfected cells expressed NFκBp65 at the same level as nontransfected samples. Next, miR-30b and miR-30c expression were validated in sham- or 2 Gy radiation with or without anti-IL-1β antibody-treated and siNFκB or control-siRNA transfected cells by quantitative real-time RT-PCR. Fig 7C shows that in the control-siRNA transfected samples, γ-radiation enhanced miR-30b and miR-30c expressions by 3- and 2.2-fold, respectively, in comparison with sham-irradiated cells. In contrast, no miR-30 increase was observed after 2 Gy irradiation to siNFκB transfected cells. It was also observed that anti-IL-1β antibody-treatment blocked the radiation-induced miR-30 expression in control-siRNA transfected cells.

## Discussion

We recently demonstrated that a naturally occurring isomer of vitamin E, delta-tocotrienol (DT3), significantly enhanced survival among mice that had received lethal doses of total-body gamma-irradiation, and protected mouse BM and GI tissue from radiation-induced damage through regulation of stress-response signal pathways involving Erk, mTOR and protein tyrosine kinase 6 [3,4]. Here we further demonstrated that DT3 at a dose of 75 mg/kg afforded a DRF of 1.28 when given 24 h before TBI, confirming its significant radioprotective efficacy. DT3 protected mouse BM hematopoietic progenitor cells and human hematopoietic stem and progenitor CD34+ cells from radiation damage, repressed the expression of radiation-induced proinflammatory factors IL-1β and IL-6 in mouse spleen, and we report for the first time that DT3 downregulated radiation-induced miR-30b and miR-30c expression in mouse tissues and serum and in human CD34+ cells.

A number of studies have examined the general and specific effects of miRNA perturbation in radiation-exposed cells and evidence for miRNA involvement in the radiation response is increasing [26–28]. We reported recently that radiation upregulates miR-30b and miR-30c in human hematopoietic CD34+ cells, and miRNA-30c plays a key role in radiation-induced human hematopoietic and osteoblast cell damage by negatively regulating survival factor REDD1 expression in these cells after γ-irradiation[19]. Due to the ability of miRNA to target multiple transcripts [29], miR-30 has been found in multiple cellular processes to regulate cell death through different genes such as cyclin D1 and D2 [30], integrin b3 (ITGB3) [31], B-Myb [32], and caspase-3 [33]. In the current study, we confirmed expression of radiation-induced miR-30b and miR-30c in mouse tissues and serum, and miR-30 expression in mouse BM, jejunum, and liver within 1 h, that returned to baseline 4 or 8 h after irradiation (data not shown). Interestingly, radiation induced miR-30 expression in serum was observed at 4 h and remained elevated up to 24 h post-irradiation. We believe that the acute secretion of extracellular miR-30 in mouse serum after radiation is likely to derive from a variety of cell types. This circulating miR-30 increase is specific, reproducible, and radiation dose-dependent in irradiated mouse serum. It is stable even after being exposed to endogenous RNase activity in serum [34–36], suggesting that miR-30 in mouse serum might be considered as a biomarker for radiation-induced pathological states. DT3 significantly suppressed miR-30 and protected animals from the acute radiation syndrome and increased survival from lethal doses of total-body irradiation. These results further support our hypothesis that levels of miR-30 in irradiated mouse tissues and serum reflect the severity of radiation damage in these animals.

In addition, we found that radiation enhanced production of pro-inflammatory cytokines IL-1β and its downstream cytokine IL-6 [37,38] in mouse spleen cells. Administration of DT3 before TBI significantly inhibited production of these cytokines. It has been suggested that release of massive radiation-induced pro-inflammatory factors from injured cells may exacerbate stress response signal activation and cell damage and depletion [5–9]. The IL-1 family of cytokines plays a key role in stress-induced acute and chronic inflammation [11,12]. Recent studies demonstrated that tocotrienols have beneficial effects including anti-cancer, anti-oxidative stress, and inhibition of nitric oxide (NO) [2,39–42]. Furthermore, DT3 is the most effective anti-inflammatory and anti-oxidative stress agent among all the vitamin E isomers tested in mouse macrophage and microglia cell lines [41,43]. Inflammatory responses play a significant role in cancer development, including the initial malignant transformation [44]. Our data suggest that the reduction of IL-1β and IL-6 after DT3 administration may contribute to protecting mouse tissues from radiation-induced inflammation and injury [45].

To further understand the interaction between miR-30 and IL-1β in response to radiation and DT3, and the mechanisms of DT3 on radiation protection, we explored the role of radiation and DT3 on regulation of miR-30 and IL-1β expression. We added IL-1β into CD34+ cell culture and observed a significant miR-30b and -30c increase in these cells (Fig 5B). Interestingly, IL-1β-induced miR-30 expression was completely blocked by DT3 treatment (Fig 5C). We further compared the effects of anti-IL-1β antibody and DT3 on miR-30 expression and survival of CD34+ cells after radiation and found that treatment with DT3 (2 μM, 24 h before irradiation) or an anti-IL-1β antibody (0.2 μg/mL, 1 h before irradiation) equally repressed expression of radiation-induced miR-30 in these cells. Both the anti-IL-1β antibody and DT3 protected against radiation-induced apoptotic cell death and increased colony formation in CD34+ cells (Fig 2C and Fig 6). These data suggest that radiation-induced IL-1β may be responsible for miR-30 expression and the radioprotective effects of DT3 may result from inhibition of a storm of radiation-induced inflammatory cytokines. We next sought to determine which a stress-response signal-transduction pathway may be involved in this IL-1β-induced miR-30 expression. To address this question, we examined the stress-response factors p53, p21 and NFκB in CD34+ cells after radiation. Our results from immunoblotting assays (Fig 7A) showed that 2 Gy γ-radiation induced p53, p21 protein expression and NFκBp65 phosphorylation in CD34+ cells. However, when CD34+ cells were treated with anti-IL-1β antibody before irradiation, the radiation-induced phosphorylation of NFκBp65 was repressed. The role of NFκB in apoptosis is complex. Both apoptosis suppression and induction have been reported [46]. It is well known that IL-1β exerts many of its biological effects by activating the transcription factor NFκB [47,48]. Here we demonstrated that IL-1β mediated NFκB activation after radiation stress. Finally, neutralization of IL-1β activation or knockdown of NFκBp65 gene expression in CD34+ cells resulted in complete abrogation of the radiation-induced miR-30 expression in these cells.

In conclusion, results from our current study demonstrated that an increase of miR-30 in irradiated cells results from a cascade of IL-1β-induced NFκB-dependent stress signals that are responsible for radiation damage in mouse and human cells. DT3 protected against radiation-induced apoptosis in mouse and human CD34+ cells through suppressing of IL-1β-induced NFκB/miR-30 signaling, and significantly enhanced survival after lethal doses of total-body γ-irradiation in mice.
